# Supplementary material for: FACT modulates the conformations of histone H2A and H2B N-terminal tails within nucleosomes
Source: Commun Biol. 2022 Aug 13;5:814. doi: 10.1038/s42003-022-03785-z (PMC9376062; doi:10.1038/s42003-022-03785-z)
Supplement: Supplementary file 2 — Supplementary information [file 42003_2022_3785_MOESM2_ESM.pdf]

Supplementary information

FACT modulates the conformations of histone H2A and H2B N-terminal tails within nucleosomes

Yasuo Tsunaka, Hideaki Ohtomo, and Yoshifumi Nishimura

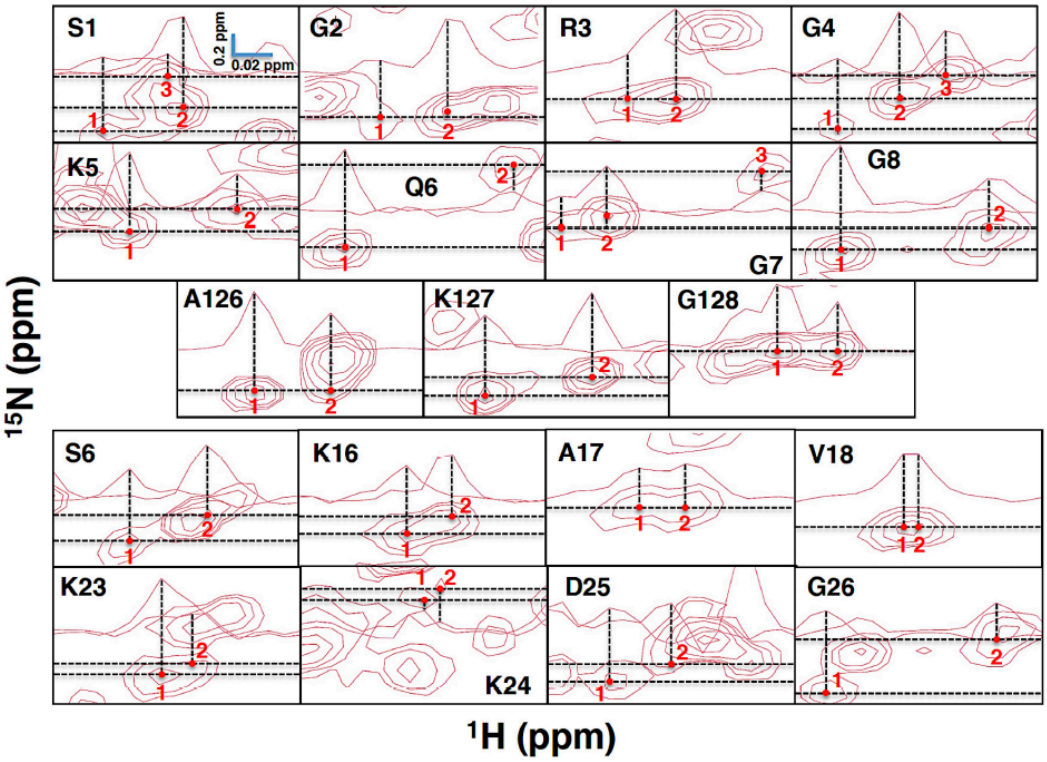

Supplementary Figure 1. Slice representations of doublet and triplet signals of the 112-bp DNA/pAID nucleosome.

NMR signals are divided into panels by amino acid residue. Filled circles and numbers indicate each signal center. One, two, or three ^{1}H spectra at the positions of the horizontal line on each ^{1}H-^{15}N spectrum are shown. Vertical lines on the ^{1}H-^{15}N spectrum indicate the peaks of the doublet or triplet signal. Signal centers are identified from peaks in the slice representations of signals.

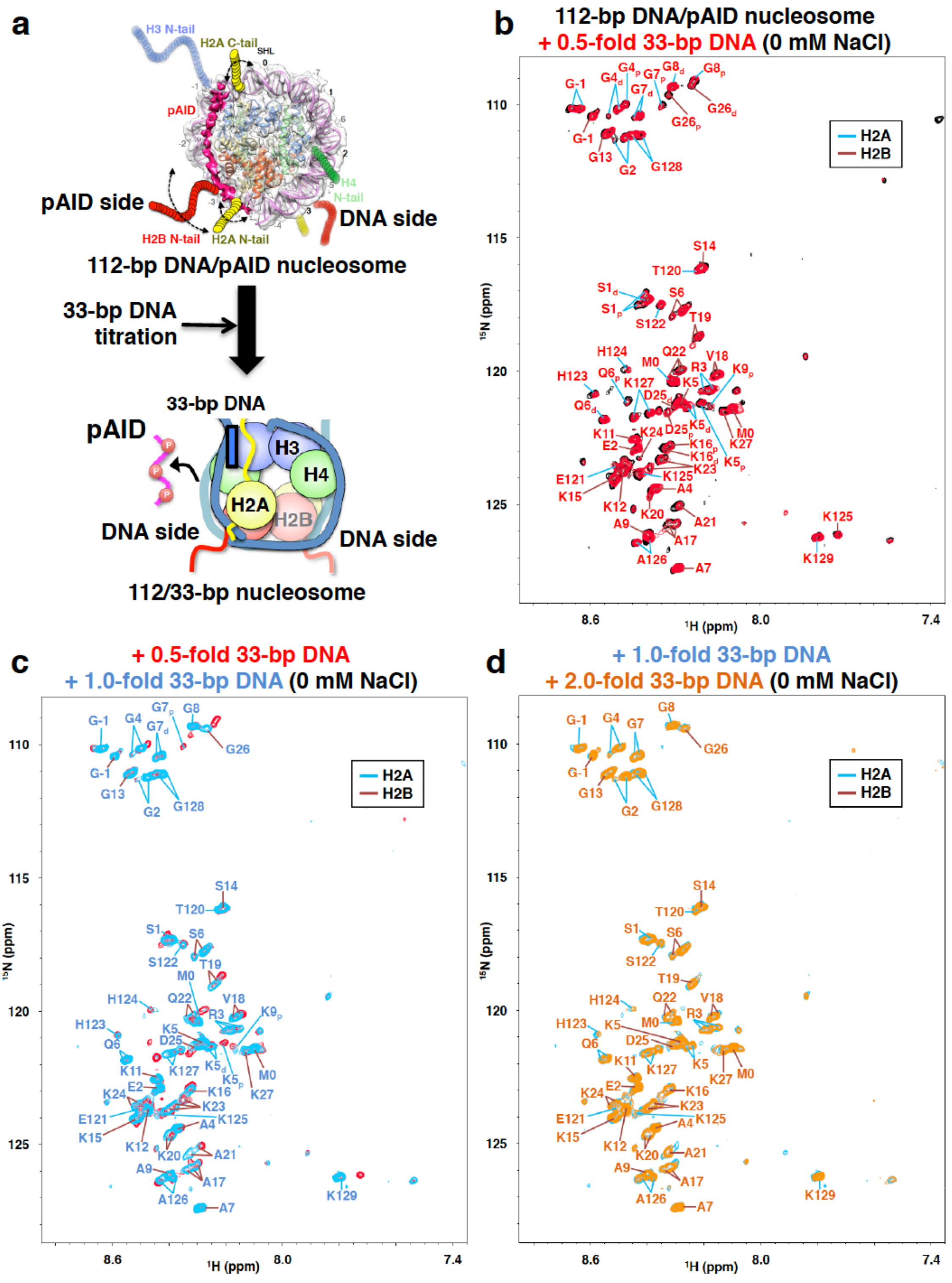

Supplementary Figure 2. 2D ^{1}H-^{15}N HSQC spectra of the H2A and H2B tails upon 33-bp DNA titration.

(a) Cartoon model of the 112/33-bp nucleosome, formed by the addition of 33-bp DNA up to an excess over the 112-bp DNA/pAID nucleosome. Cryo-EM

structure of the 112-bp DNA/pAID nucleosome is shown as in Fig. 1a. Histone proteins and DNA are colored as follows: H2A (yellow), H2B (red), H3 (light blue), H4 (green), and DNA (light sea green). The H2A and H2B N-tails are indicated by yellow and red strings, respectively. pAID is colored magenta. Red circles labeled with P indicate phosphorylation.

(b) Spectral superposition of the 2D ^{1}H-^{15}N HSQC spectra of the 112-bp DNA/pAID nucleosome without (black) and with the addition of 33-bp DNA at a molar ratio of 1:0.5 (red). Signal assignments in the 112-bp DNA/pAID nucleosome upon titration with 33-bp DNA at a molar ratio of 1:0.5 are labeled in red. pAID and DNA side signals are designated by subscript p and d, respectively. Blue and orange lines indicate residues of H2A and H2B, respectively.

(c) Spectral superposition of the 2D ^{1}H-^{15}N HSQC spectra of the 112-bp DNA/pAID nucleosome upon titration with 33-bp DNA at molar ratios of 1:0.5 (red) and 1:1 (cyan). Signal assignments in the 112-bp DNA/pAID nucleosome upon an equivalent addition of 33-bp DNA are labeled in cyan.

(d) Spectral superposition of the 2D ^{1}H-^{15}N HSQC spectra of the 112-bp DNA/pAID nucleosome upon titration with 33-bp DNA at molar ratios of 1:1 (cyan) and 1:2 (orange). Signal assignments in the 112-bp DNA/pAID nucleosome upon two-fold addition of 33-bp DNA are labeled in orange.

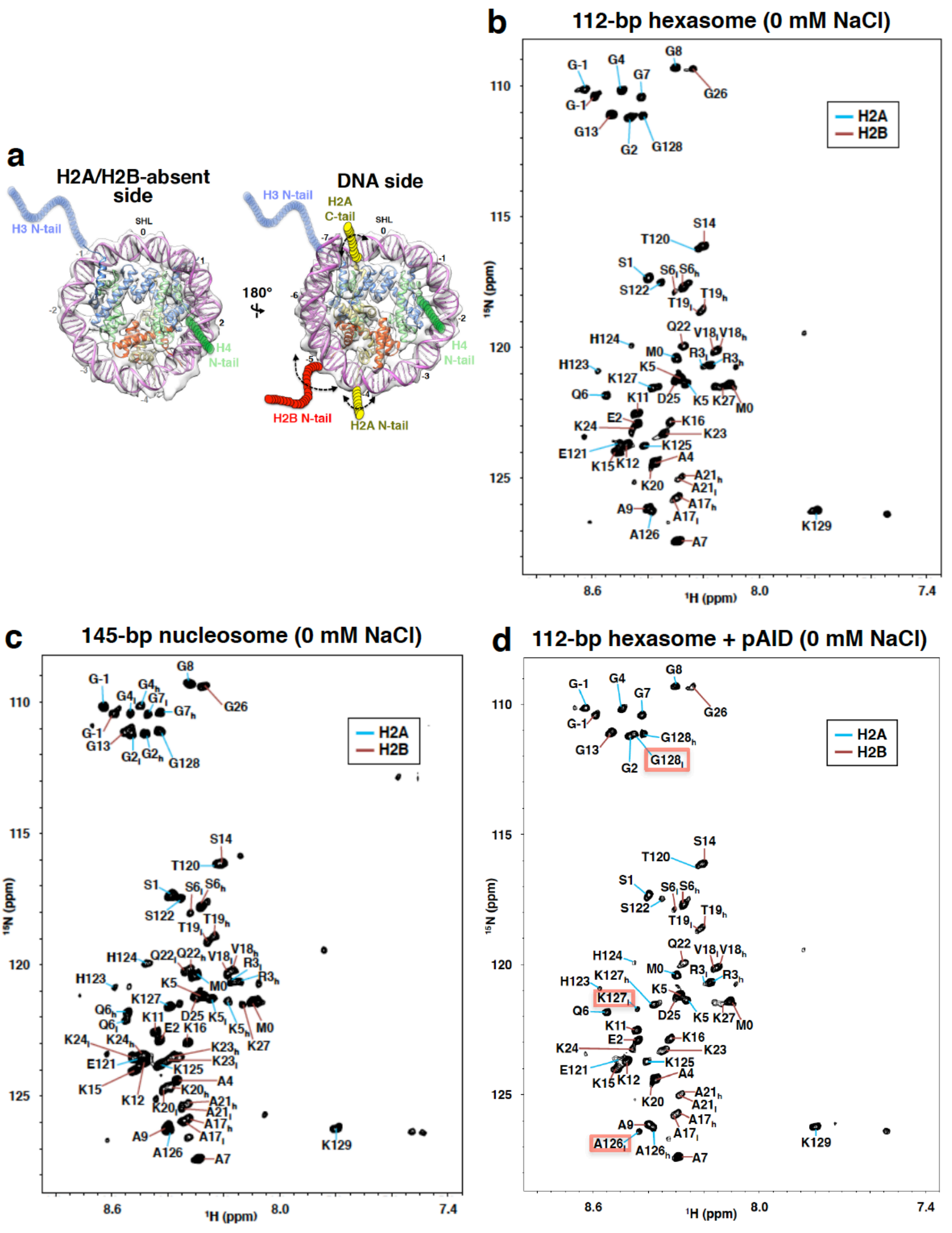

Supplementary Figure 3. 2D ^{1}H-^{15}N HSQC spectra of the H2A and H2B tails in the 112-bp hexasome and 145-bp nucleosome.

(a) Cryo-EM structure of the 112-bp hexasome. Two views of the EM density map of the 112-bp hexasome (EMD-6939) are superimposed on the nucleosome structure (PDB ID: 2CV5) lacking the 43-bp DNA and one

H2A/H2B dimer, colored as in Fig. 1 and generated by UCSF Chimera[67]. Colored circular chains denote individual histone tails, which are not observed in the EM structure. Superhelix locations (SHL), representing the number of double turns from the dyad axis of the canonical nucleosome (0), are indicated.

(b, c, d) 2D ^{1}H-^{15}N HSQC spectra of the 112-bp hexasome (b), 145-bp nucleosome (c), and 112-bp hexasome upon the addition of an equivalent amount of pAID (d) recorded in 20 mM HEPES-NaOH, pH 7.0, 10% D_2O on a 950-MHz spectrometer. High- and low-field components of doublet signals are designated by subscript h and l, respectively. Blue and orange lines indicate residues of H2A and H2B, respectively. Red boxes indicate signals observed after the addition of pAID.

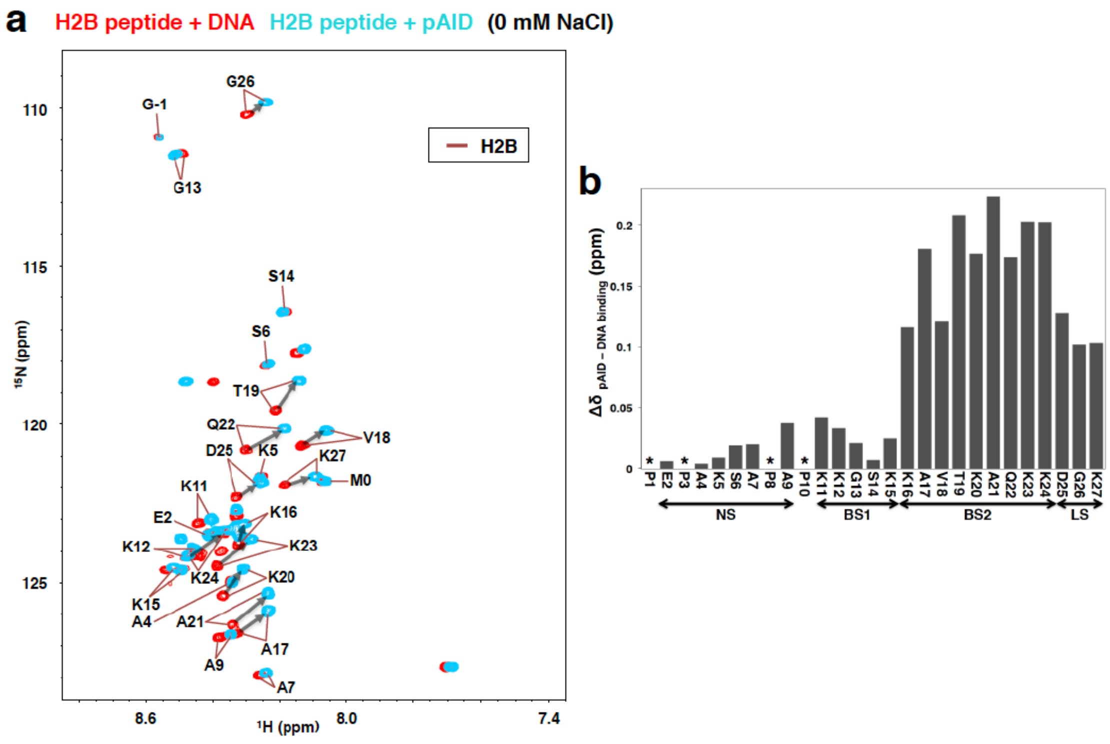

Supplementary Figure 4. Conformational comparison between pAID- and DNA-bound H2B peptides.

(a) Comparison of 2D ^{1}H-^{15}N HSQC spectra of the H2B peptide upon the addition of an equivalent amount of DNA (red) or pAID (cyan) recorded on a 600-MHz spectrometer. To aid visualization, arrows connect the DNA-bound signals to the pAID-bound signals for Lys16–Lys27.

(b) Histogram showing chemical shift differences between the pAID- and DNA-bound H2B peptides. Chemical shift differences are plotted for each residue of the H2B N-tail. Asterisks (*) indicate residues that were either not observed or not assigned.

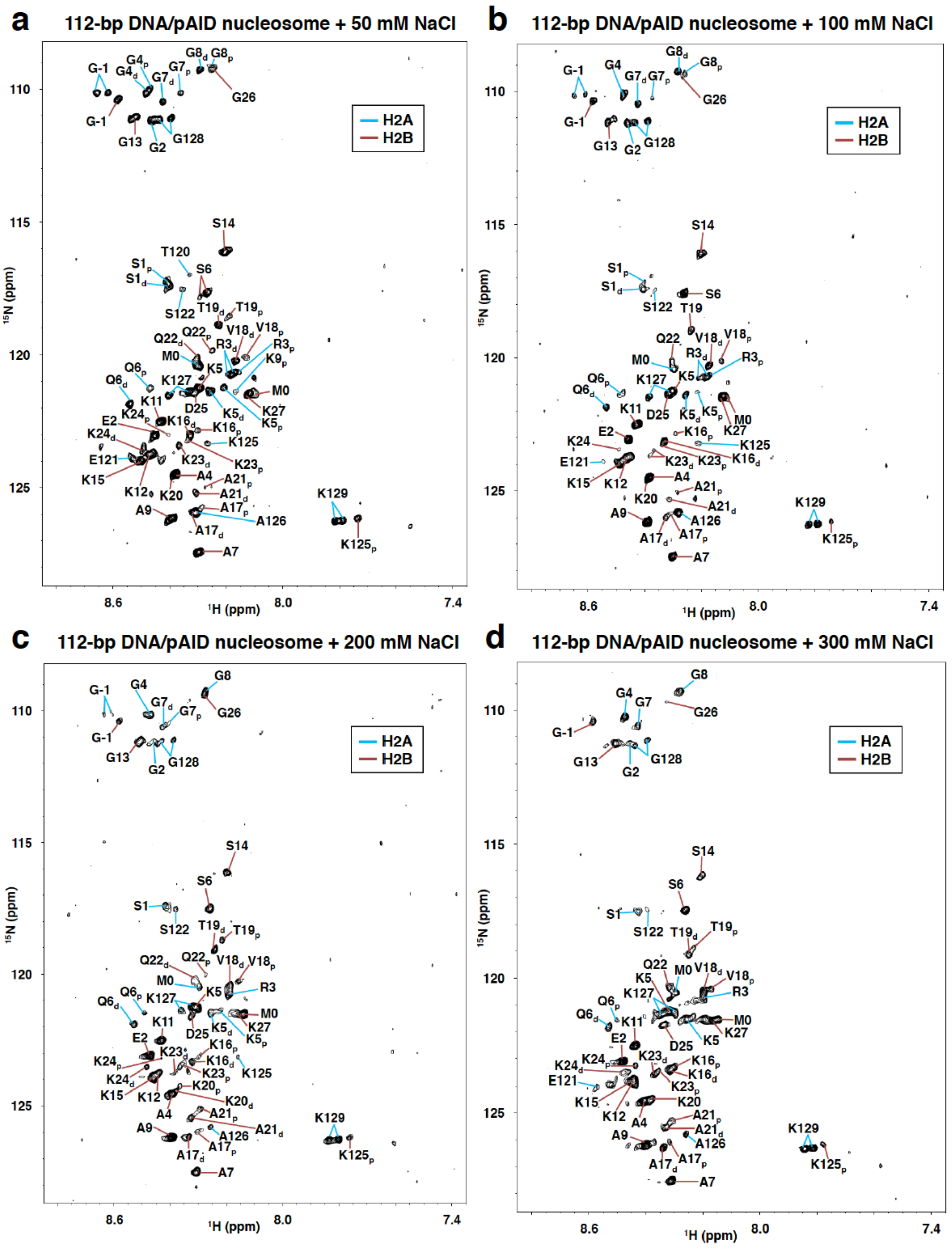

Supplementary Figure 5. NMR spectra of the 112-bp DNA/pAID nucleosome upon NaCl titration.

(a, b, c, d) 2D ^{1}H-^{15}N HSQC spectra of the 112-bp DNA/pAID nucleosome recorded on a 950-MHz spectrometer upon NaCl titration at 50 mM (a), 100 mM (b), 200 mM (c), and 300 mM (d). Blue and orange lines indicate residues

of H2A and H2B, respectively. pAID and DNA side signals are designated by subscript p and d, respectively.

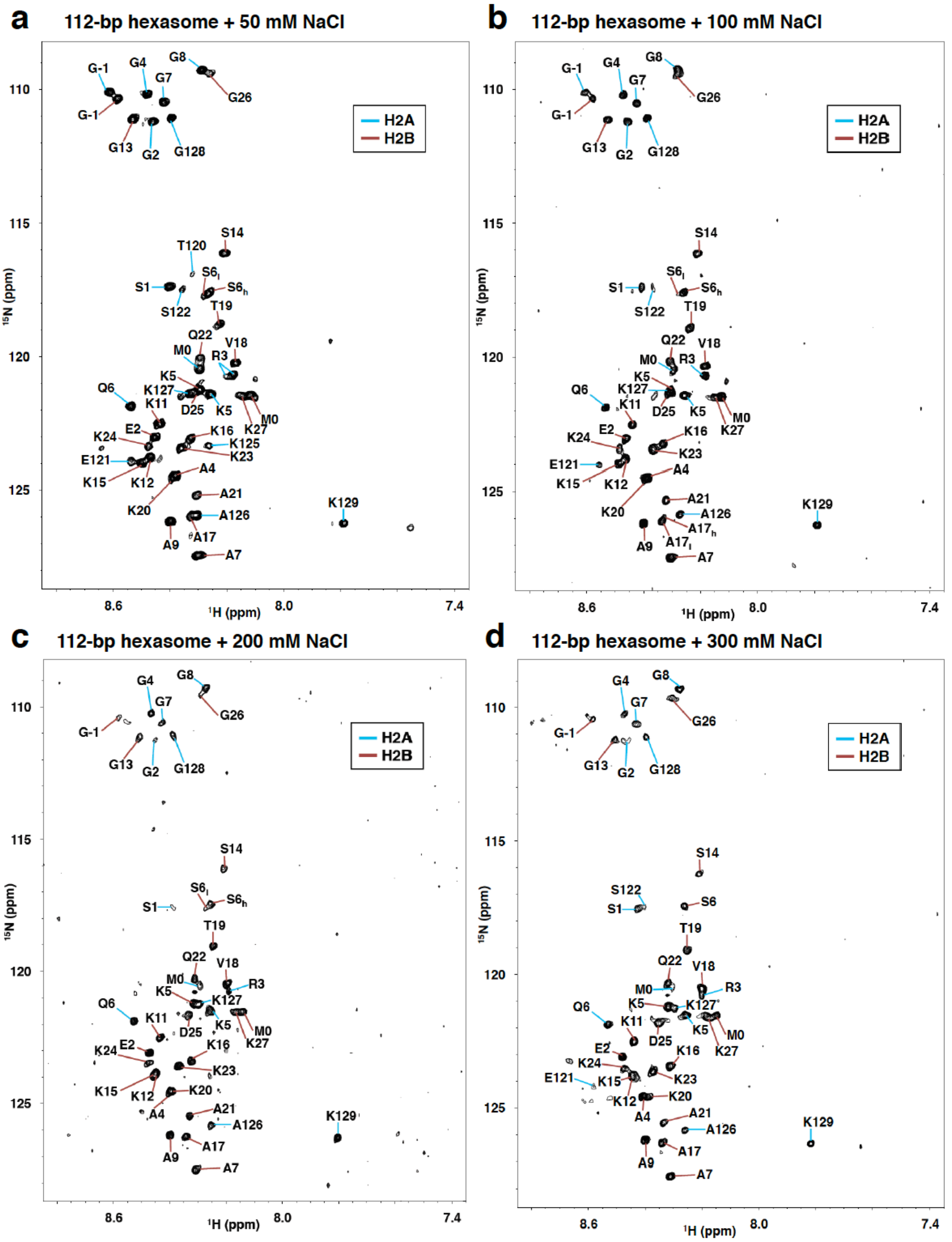

Supplementary Figure 6. NMR spectra of the 112-bp hexasome upon NaCl titration.

(a, b, c, d) 2D ^{1}H-^{15}N HSQC spectra of the 112-bp hexasome recorded on a 950-MHz spectrometer upon NaCl titration at 50 mM (a), 100 mM (b), 200 mM (c), and 300 mM (d). Blue and orange lines indicate residues of H2A and

H2B, respectively. High- and low-field components of doublet signals are designated by subscript h and l, respectively.

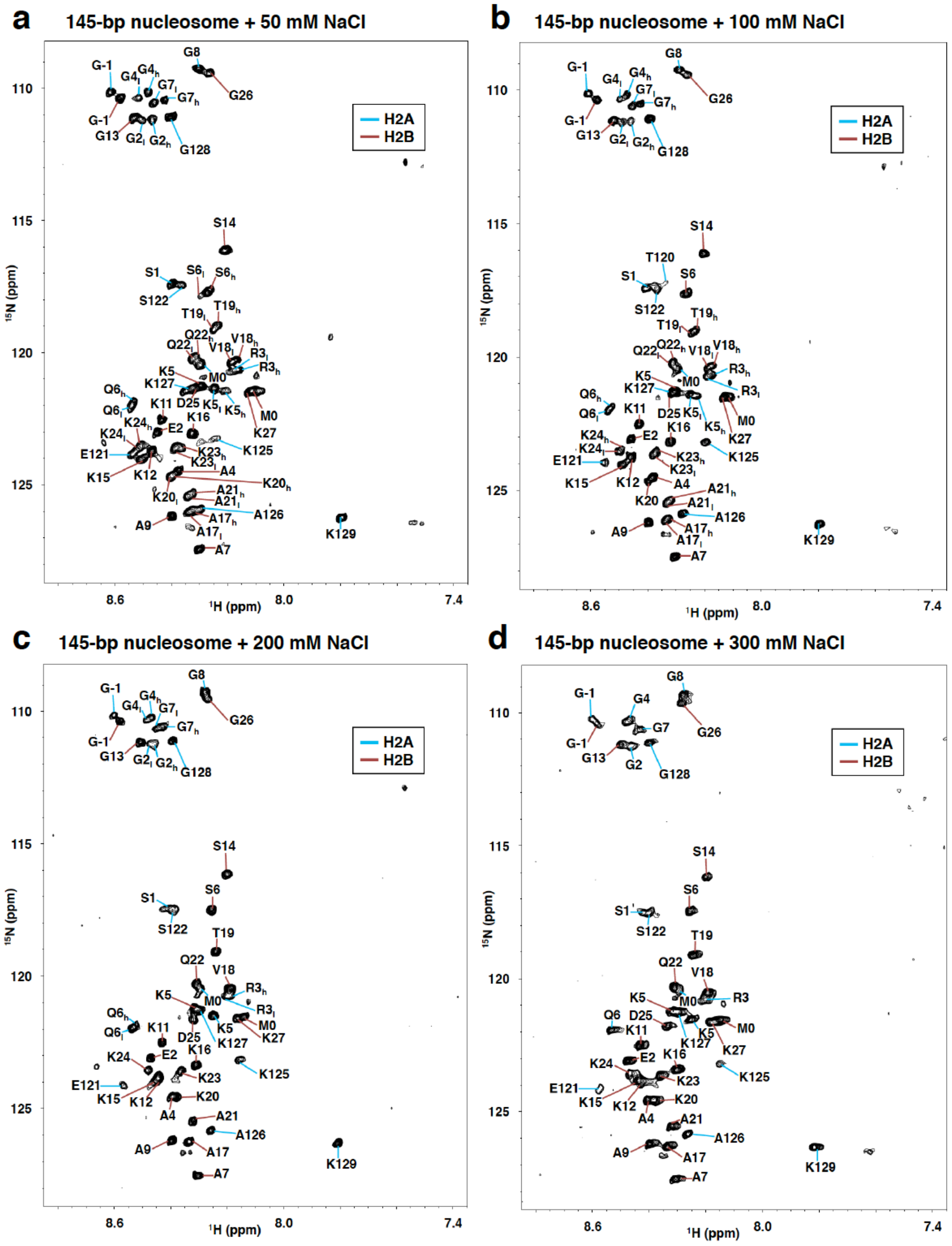

Supplementary Figure 7. NMR spectra of the 145-bp nucleosome upon NaCl titration.

(a, b, c, d) 2D ^{1}H-^{15}N HSQC spectra of the 145-bp nucleosome recorded on a 950-MHz spectrometer upon NaCl titration at 50 mM (a), 100 mM (b), 200 mM (c), and 300 mM (d). Blue and orange lines indicate residues of H2A and

H2B, respectively. High- and low-field components of doublet signals are designated by subscript h and l, respectively.

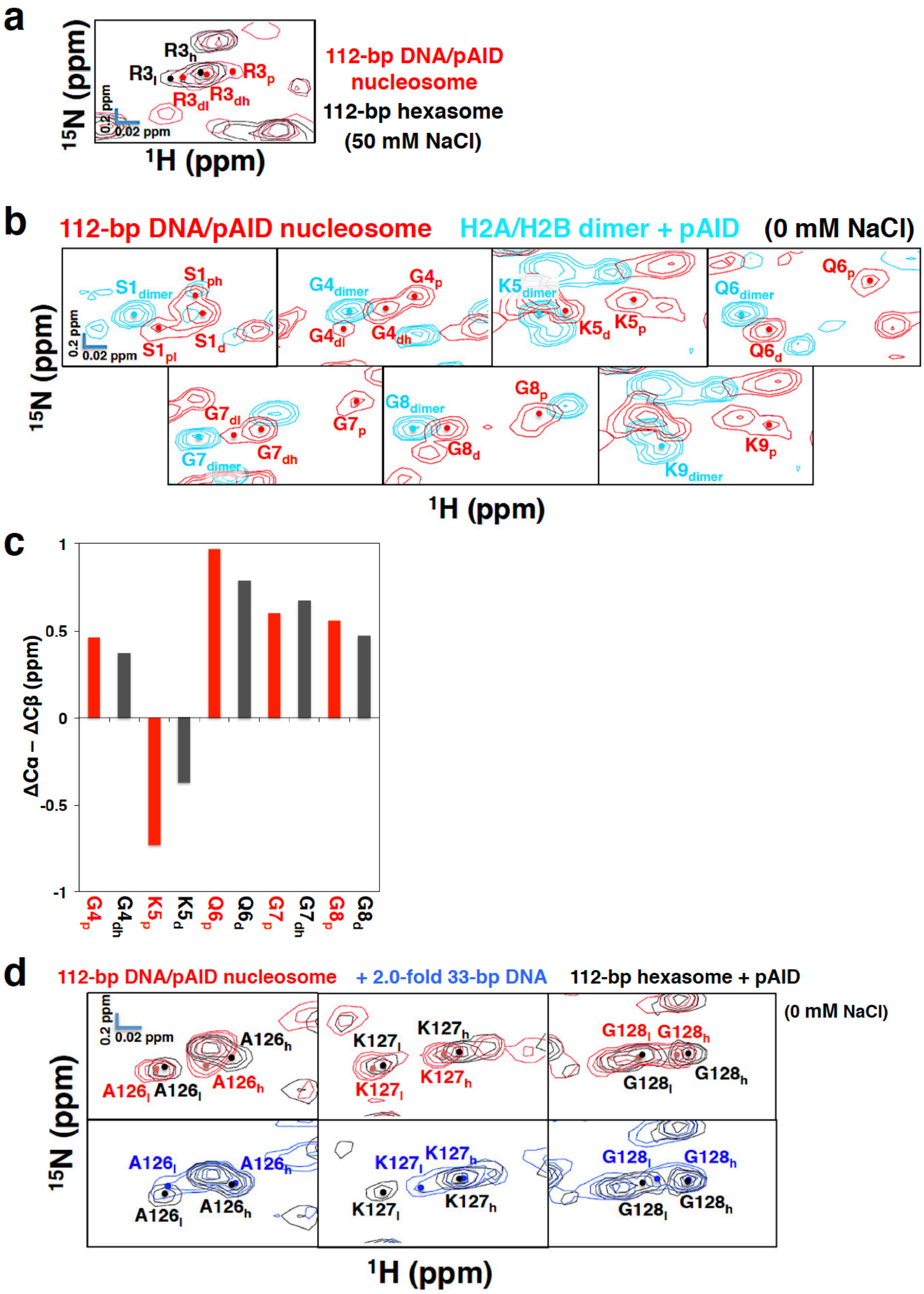

Supplementary Figure 8. Comparison of chemical environments around the H2A tails in various complexes.

(a) Expanded signal comparison of Arg3 in the H2A N-tails between the 112-bp DNA/pAID nucleosome (red) and the 112-bp hexasome (black) at 50 mM NaCl. Filled circles indicate each signal center. pAID and DNA side

signals are designated by subscript p and d, respectively. High- and low-field components of signals are designated by subscript h and l, respectively.

(b) Expanded signal comparison of the H2A N-tails between the 112-bp DNA/pAID nucleosome (red) and H2A/H2B dimer upon the addition of an equivalent amount of pAID (cyan) at 0 mM NaCl. NMR signals are divided into panels by amino acid residue. Filled circles indicate each signal center. Signals corresponding to the 112-bp DNA/pAID nucleosome are labeled in red; those corresponding to the H2A/H2B dimer after pAID addition are labeled in cyan with the subscript "dimer".

(c) Chemical shift index of the H2A N-tail on the pAID or DNA side in the 112-bp DNA/pAID nucleosome. "$\Delta C\alpha - \Delta C\beta$" values of pAID side (red) and DNA side (black) residues are shown.

(d) Expanded signal comparison of Ala126–Gly128 in H2A C-tails between the 112-bp hexasome upon the addition of an equivalent amount of pAID (black) and the 112-bp DNA/pAID nucleosome alone (red) (upper panel) or with the two-fold addition of 33-bp DNA (112/33-bp nucleosome, blue) (lower panel). NMR signals are divided into panels by amino acid residue. Filled circles indicate each signal center.

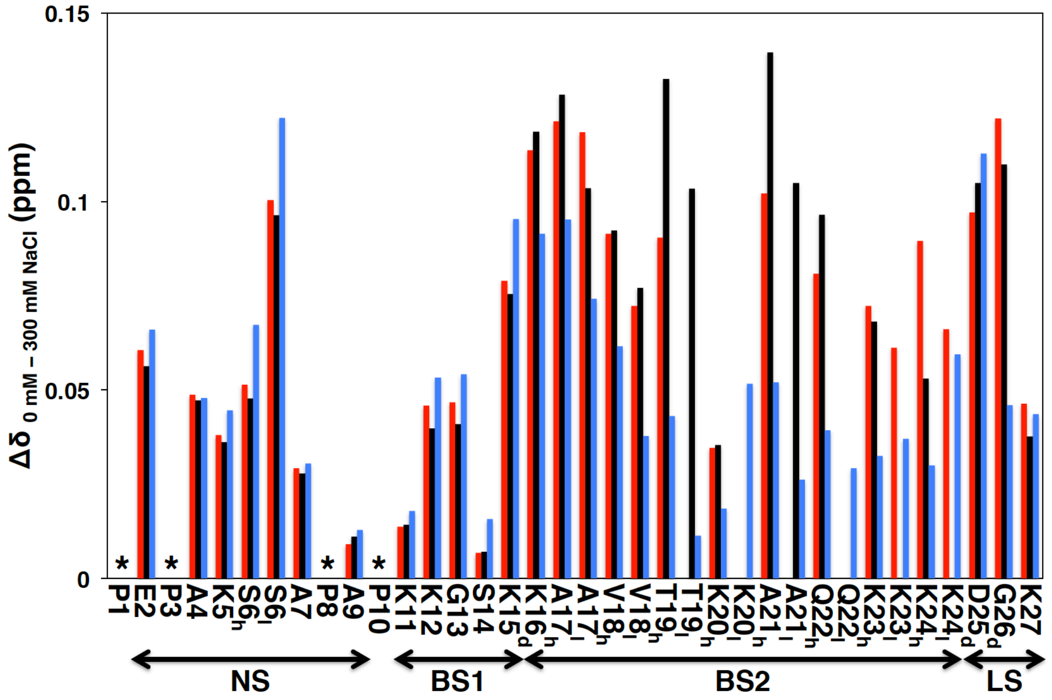

Supplementary Figure 9. Histogram showing chemical shift differences between 0 and 300 mM NaCl for residues of the DNA side H2B N-tail in the 112-bp DNA/pAID nucleosome (red), the 112-bp hexasome (black), and the 145-bp nucleosome (blue). Chemical shift differences are plotted for each signal of the DNA side residues. Asterisks (*) indicate residues that were either not observed or not assigned.

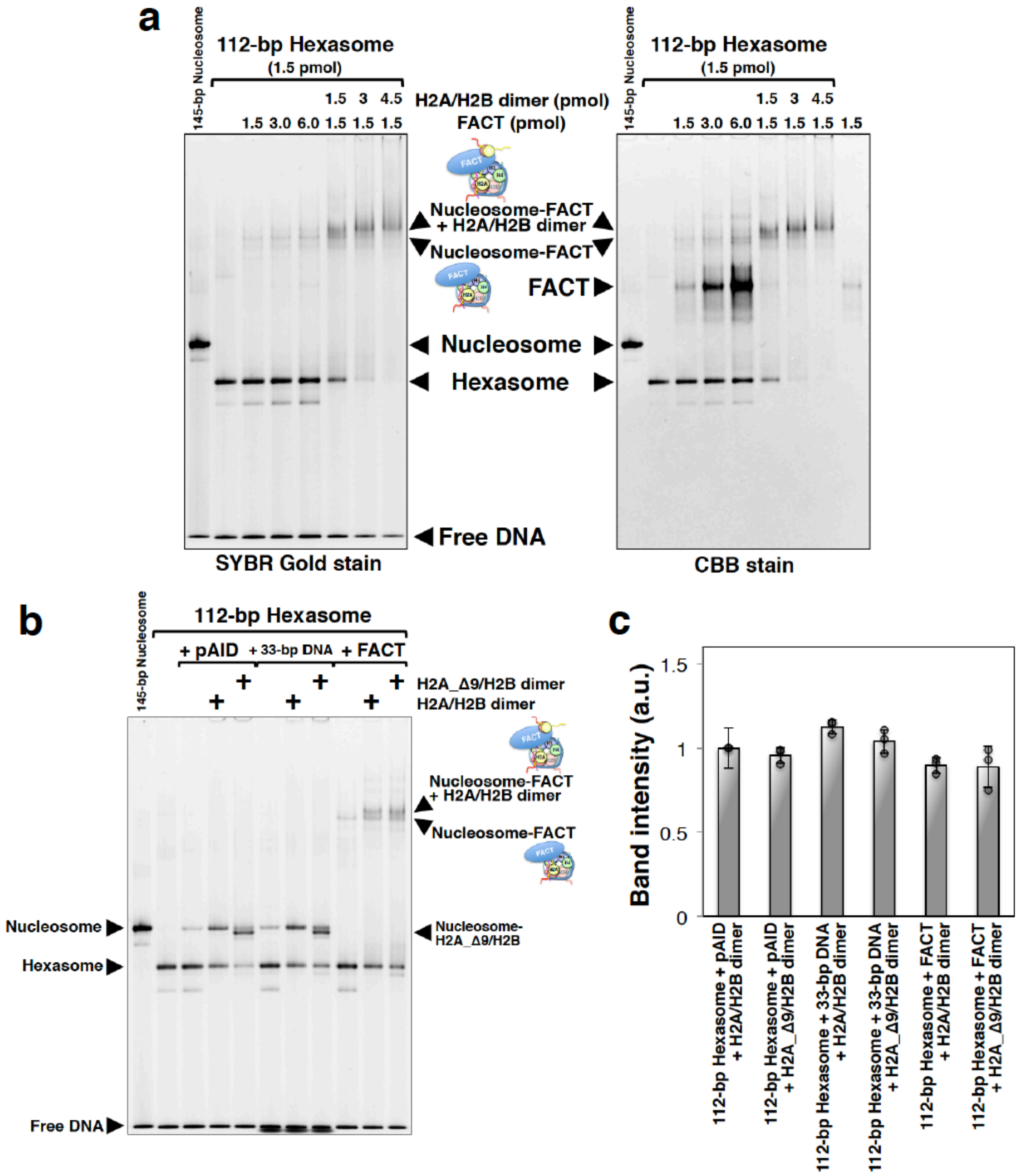

Supplementary Figure 10. EMSAs of nucleosome assembly with FACT.

(a) Representative SYBR Gold-stained (left) and CBB-stained (right) EMSAs of nucleosomal complexes in different mixtures. The cartoon model of two nucleosomal complexes with FACT is shown as in Fig. 6a. Experiments were repeated at least three times. The full gel image is shown in Supplementary Fig. 13. For the 1:1 mixture of FACT and the 112-bp hexasome, the CBB-stained gel detected two main bands and a slower-migrating faint band

corresponding to the 112-bp hexasome, FACT, and the complex with FACT, respectively. The hexasome band and the faint band of the complex were hardly changed upon the excess addition of FACT, indicating that FACT hardly binds to the 112-bp hexasome. One the other hand, for the 1:1:1 mixture of the 112-bp hexasome, FACT, and the H2A/H2B dimer, the band intensities of hexasome and FACT were significantly reduced, while two bands of complexes with FACT were more obvious. Upon the two-fold addition of H2A/H2B dimer to the 112-bp hexasome and FACT, the two bands corresponding to the nucleosomal complexes with FACT were clearly joined to the slower band, which is regarded as the nucleosome-FACT complex with the additional H2A/H2B dimer. Taken together, the two bands are the nucleosome-FACT and nucleosome-FACT-H2A/H2B complexes, respectively. Therefore, the band intensity of the nucleosomal complexes with FACT shown in Fig. 6c and Supplementary Fig. 10c were derived from the bands of both complexes.

(b) Representative SYBR Gold-stained EMSAs of nucleosomal complexes in different mixtures. Experiments were repeated at least three times. The full gel image is shown in Supplementary Fig. 13.

(c) Band intensity of each nucleosomal complex in panel (b). Band intensity was quantified by using Image Lab software (Bio-Rad), and the intensity of the 112-bp hexasome with pAID and H2A/H2B dimer was set to one. Averages from at least three independent experiments are shown; error bars represent SD.

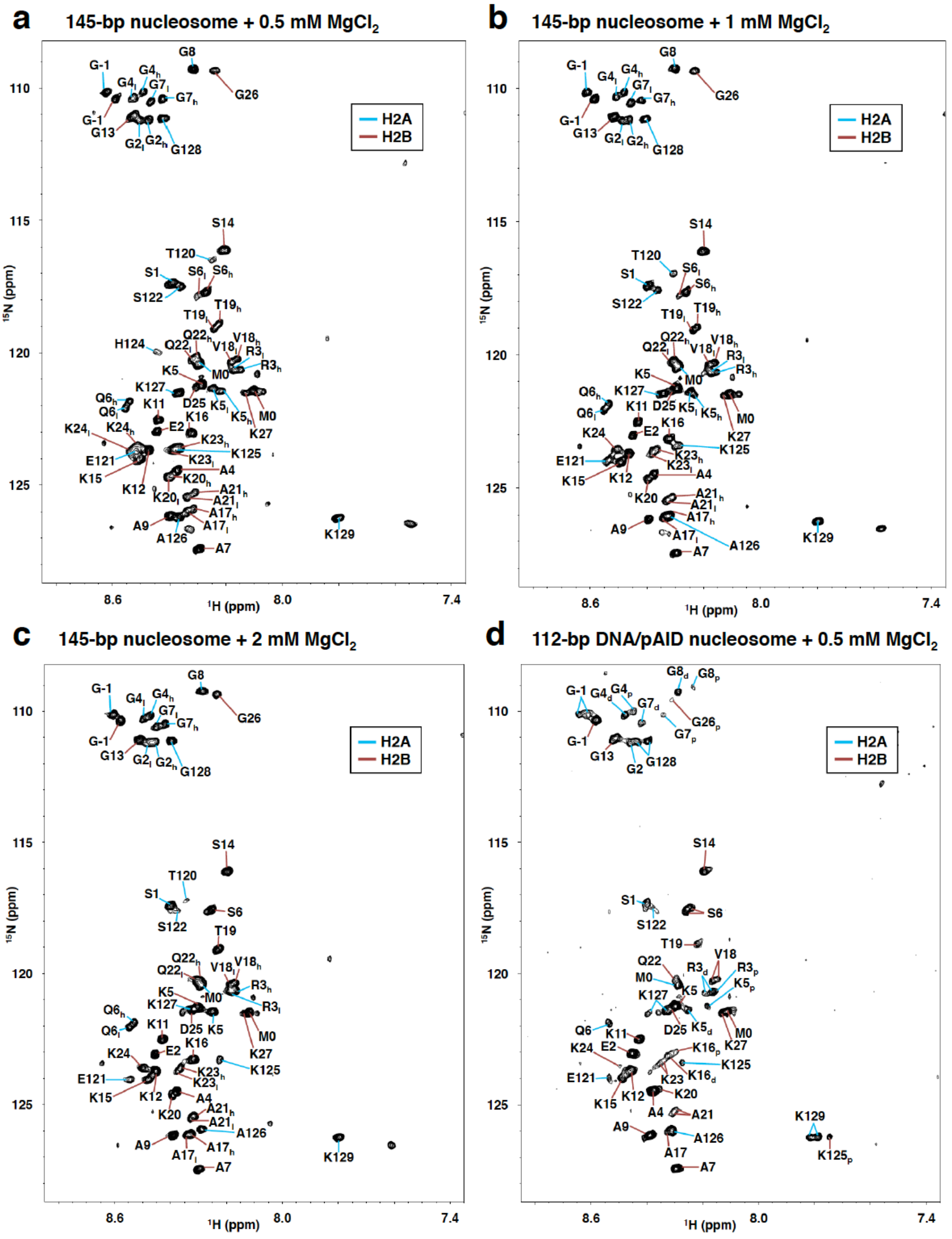

Supplementary Figure 11. NMR spectra of the 112-bp DNA/pAID nucleosome and 145-bp nucleosome upon $MgCl_2$ titration.

(a, b, c) 2D ^{1}H-^{15}N HSQC spectra of the 145-bp nucleosome recorded on a 950-MHz spectrometer upon $MgCl_2$ titration at 0.5 mM (a), 1 mM (b), and 2 mM (c). Blue and orange lines indicate residues of H2A and H2B,

respectively. High- and low-field components of signals are designated by subscript h and l, respectively.

(d) 2D ^{1}H-^{15}N HSQC spectrum of the 112-bp DNA/pAID nucleosome recorded on a 950-MHz spectrometer at 0.5 mM $MgCl_2$. pAID and DNA side signals are designated by subscript p and d, respectively.

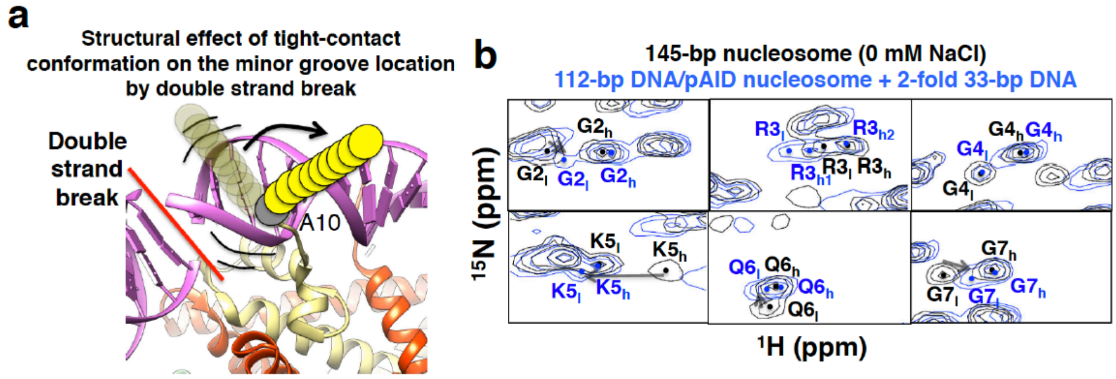

Supplementary Figure 12. Conformational comparison of the H2A and H2B tails in nucleosome.

(a) Schematic view of the dynamic structure of the proximal H2A N-tail around the double-strand break in the 112/33-bp nucleosome. The structural model of the 112/33-bp nucleosome is built on the nucleosome structure (PDB ID: 2CV5), colored as in Fig. 1. Yellow circular chains denote the H2A N-tails. Arrow represents the dynamic behavior of the H2A N-tail.

(b) Expanded signal comparison of Gly2–Gly7 in the H2A N-tail between the 145-bp nucleosome (black) and the two-fold addition of 33-bp DNA to the 112-bp DNA/pAID nucleosome (112/33-bp nucleosome, blue) at 0 mM NaCl. NMR signals are divided into panels by amino acid residue. Signal assignments in the 112/33-bp nucleosome and 145-bp nucleosome are labeled in blue and black, respectively. Filled circles indicate each signal center. High- and low-field components of signals are designated by subscript h and l, respectively. To aid visualization, arrows connect signals of the contact conformation of the 145-bp nucleosome ($G2_l$, $K5_h$, $Q6_l$, and $G7_l$) to the corresponding signals of the 112/33-bp nucleosome ($G2_l$, $K5_l$, $Q6_l$, and $G7_l$).

Supplementary Fig. 7a (SYBR Gold stain)

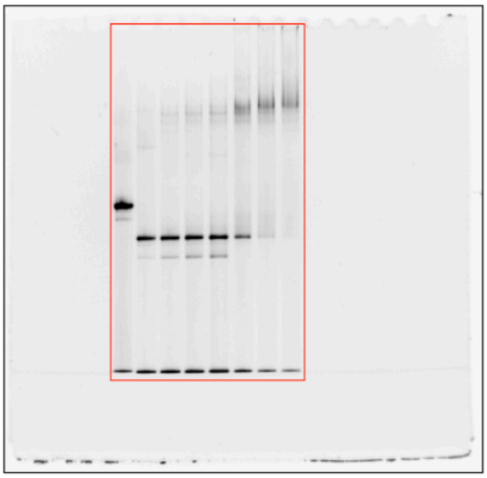

Supplementary Fig. 7a (CBB stain)

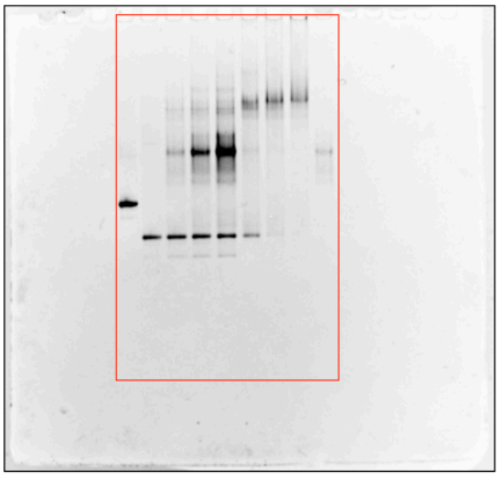

Fig. 6b

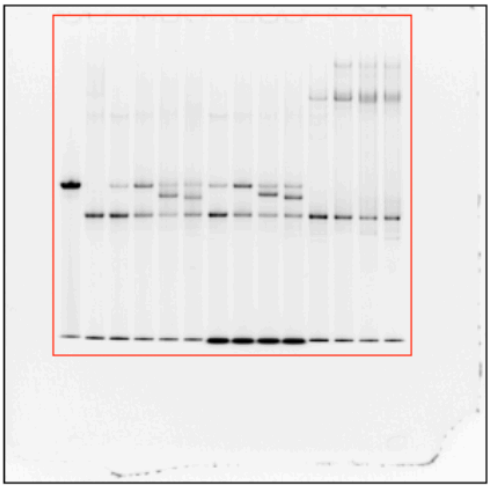

Supplementary Fig. 7b

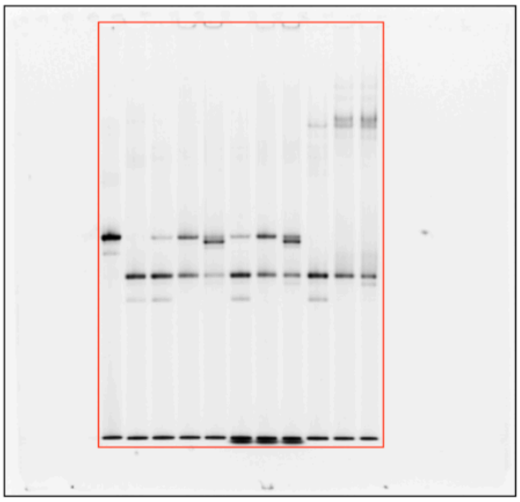

Supplementary Figure 13. Full gel image of EMSAs.

Supplementary Table 1. Summary of the observed signal changes of H2A in the NMR spectra of different complexes at 100 mM NaCl. Corresponding signals in each nucleosomal complex are displayed side by side.

H2A residues		112-bp DNA/pAID nucleosome	145-bp nucleosome	112-bp hexasome	Contact state
N-tail	Ser1	$S1_p$	-	-	pAID
		$S1_d$	S1	S1	DNA
	Gly2	G2	$G2_h$	G2	Reduced DNA
		-	$G2_l$	-	DNA
	Arg3	$R3_p$	-	-	pAID
		$R3_{dh}$	$R3_h$	R3	DNA
		$R3_{dl}$	$R3_l$: Shift	-	DNA
	Gly4	G4	$G4_h$	G4	Reduced DNA
		-	$G4_l$	-	DNA
	Lys5	$K5_p$	-	-	pAID
		-	$K5_h$	-	DNA
		$K5_d$	$K5_l$	K5	Reduced DNA
	Gln6	$Q6_p$	-	-	pAID
		$Q6_d$	$Q6_h$	Q6	Reduced DNA
		-	$Q6_l$	-	DNA
	Gly7	$G7_p$	-	-	pAID
		$G7_d$	$G7_h$	G7	Reduced DNA
		-	$G7_l$	-	DNA
	Gly8	$G8_p$	-	-	pAID
		$G8_d$	G8	G8	DNA
C-tail	Thr120	-	T120	-	-
	Glu121	E121	E121	E121	DNA/pAID
	Ser122	S122	S122	S122	DNA/pAID
	His123	-	-	-	-
	His124	-	-	-	-
	Lys125	K125	K125	K125	DNA/pAID
	Ala126	A126	A126	A126	DNA/pAID
	Lys127	$K127_h$	K127	K127	DNA/pAID
		$K127_l$	-	-	Free pAID
	Gly128	$G128_h$	G128	G128	DNA/pAID
		$G128_l$	-	-	Free pAID
	Lys129	$K129_h$	K129	K129	DNA/pAID
		$K129_l$	-	-	Free pAID

Supplementary Table 2. Summary of the observed signal changes of H2B in the NMR spectra of different complexes at 100 mM NaCl. Corresponding signals in each nucleosomal complex are displayed side by side.

H2B residues		112-bp DNA/pAID nucleosome	145-bp nucleosome	112-bp hexasome	Contact state
LS	Pro1	-	-	-	-
	Glu2	E2	E2	E2	DNA/pAID
	Pro3	-	-	-	-
	Ala4	A4	A4	A4	DNA/pAID
	Lys5	K5	K5	K5	DNA/pAID
	Ser6	S6	S6	$S6_h$	DNA/pAID
		-	-	$S6_l$	DNA/pAID
	Ala7	A7	A7	A7	DNA/pAID
	Pro8	-	-	-	-
	Ala9	A9	A9	A9	DNA/pAID
	Pro10	-	-	-	-
BS1	Lys11	K11	K11	K11	DNA/pAID
	Lys12	K12	K12	K12	DNA/pAID
	Gly13	G13	G13	G13	DNA/pAID
	Ser14	S14	S14	S14	DNA/pAID
	Lys15	K15	K15	K15	DNA/pAID
BS2	Lys16	$K16_p$	-	-	pAID
		$K16_d$	K16	K16	DNA
	Ala17	$A17_p$	-	-	pAID
		$A17_d$	$A17_h$	$A17_h$	DNA
		-	$A17_l$	$A17_l$	Reduced DNA
	Val18	$V18_p$	-	-	pAID
		$V18_d$	$V18_h$	V18	DNA
		-	$V18_l$	-	Reduced DNA
	Thr19	T19	$T19_h$	T19	DNA
		-	$T19_l$	-	Reduced DNA
	Lys20	K20	K20	K20	DNA
	Ala21	$A21_p$	-	-	pAID
		$A21_d$	$A21_h$	A21	DNA
		-	$A21_l$	-	Reduced DNA
	Gln22	Q22	$Q22_h$	Q22	DNA
		-	$Q22_l$	-	Reduced DNA
	Lys23	$K23_p$	-	-	pAID
		$K23_{dh}$	$K23_h$	K23	DNA
		$K23_{dl}$	$K23_l$	-	DNA
	Lys24	K24	$K24_h$	K24	DNA
		-	$K24_l$	-	Reduced DNA
LS	Asp25	D25	D25	D25	DNA/pAID
	Gly26	G26	G26	G26	DNA/pAID
	Lys27	K27	K27	K27	DNA/pAID
Lys125		$K125_p$	-	-	pAID
