# Supplementary material for: FACT modulates the conformations of histone H2A and H2B N-terminal tails within nucleosomes
Source: Commun Biol. 2022 Aug 13;5:814. doi: 10.1038/s42003-022-03785-z (PMC9376062; doi:10.1038/s42003-022-03785-z)
Supplement: Supplementary file 3 — Description of Additional Supplementary Files [file 42003_2022_3785_MOESM3_ESM.pdf]

Description of Additional Supplementary Files

File name: Supplementary Data 1
Description: The source data in Figs. 4d, 5c, and 6c.
